# Preserved Crossmodal Integration of Emotional Signals in Binge Drinking

**DOI:** 10.3389/fpsyg.2017.00984

**Published:** 2017-06-15

**Authors:** Séverine Lannoy, Valérie Dormal, Mélanie Brion, Joël Billieux, Pierre Maurage

**Affiliations:** ^1^Laboratory for Experimental Psychopathology, Psychological Science Research Institute, Université catholique de LouvainLouvain-la-Neuve, Belgium; ^2^Institute for Health and Behavior, Integrative Research Unit on Social and Individual Development, University of LuxembourgEsch-sur-Alzette, Luxembourg

**Keywords:** heavy drinking, emotion, facial expression, prosody, alcohol-dependence

## Abstract

Binge drinking is an alcohol consumption pattern with various psychological and cognitive consequences. As binge drinking showed qualitatively comparable cognitive impairments to those reported in alcohol-dependence, a continuum hypothesis suggests that this habit would be a first step toward alcohol-related disorders. Besides these cognitive impairments, alcohol-dependence is also characterized by large-scale deficits in emotional processing, particularly in crossmodal contexts, and these abilities have scarcely been explored in binge drinking. Emotional decoding, most often based on multiple modalities (e.g., facial expression, prosody or gesture), yet represents a crucial ability for efficient interpersonal communication and social integration. The present study is the first exploration of crossmodal emotional processing in binge drinking, in order to test whether binge drinkers already present the emotional impairments described among alcohol-dependent patients, in line with the continuum hypothesis. Twenty binge drinkers and 20 matched controls performed an experimental task requiring the identification of two emotions (happiness or anger) presented in two modalities (visual or auditory) within three conditions (unimodal, crossmodal congruent or crossmodal incongruent). In accordance with previous research in binge drinking and alcohol-dependence, this study was based on two main hypotheses. First, binge drinkers would present a reduced facilitation effect (i.e., classically indexed in healthy populations by faster reaction times when two congruent modalities are presented simultaneously). Second, binge drinkers would have higher difficulties to inhibit interference in incongruent modalities. Results showed no significant difference between groups in emotional decoding ability, whatever the modality or condition. Control participants, however, appeared slower than binge drinkers in recognizing facial expressions, also leading to a stronger facilitation effect when the two modalities were presented simultaneously. However, findings did not show a disrupted facilitation effect in binge drinkers, whom also presented preserved performance to inhibit incongruence during emotional decoding. The current results thus suggest that binge drinkers do not demonstrate a deficit for emotional processing, both in unimodal and crossmodal contexts. These results imply that binge drinking might not be characterized by impairments for the identification of primary emotions, which could also indicate that these emotional processing abilities are well-preserved at early stages of excessive alcohol consumption.

## Introduction

Excessive alcohol consumption represents a major public health problem, directly involved in 4% of deaths worldwide (Rehm et al., 2009), and is also considered as a major concern in adolescents and young adults. Indeed, binge drinking, defined as the consumption of at least 4 (for women) or 5 (for men) drinks within 2 hours (i.e., representing a blood concentration of 0.08 g/dl) (National Institute of Alcohol Abuse and Alcoholism [NIAAA], 2004) has become widespread in this population. While this NIAAA definition is the most reported in the exploration of binge drinking habits, studies currently use various ways to identify binge drinkers. To date, the main categorizations are the self-reported number of alcohol drinks consumed per occasion (e.g., Keller et al., 2007), with different levels of frequency, and the computation of a binge drinking score based on the self-described consumption speed and drunkenness episodes (Townshend and Duka, 2005). As a whole, this topic has recently led to increasing research showing that binge drinking is associated with a large range of consequences. First, at short term, binge drinkers are exposed to higher dangerous issues, such as hypothermia, or risks for falling or drowning (Hingson et al., 2009). Importantly, from a cognitive view and in a longer term perspective, binge drinking is also characterized by reduced performance in memory, executive or attentional abilities. Several subcomponents of memory, like spatial, declarative, episodic, and prospective memory, appear impaired in binge drinkers (e.g., Hartley et al., 2004; Heffernan et al., 2010; Heffernan and O’Neill, 2012). Concerning executive functions, findings indicated slower planning (Hartley et al., 2004), disadvantageous decision-making (e.g., Goudriaan et al., 2007), as well as impaired post-error slowing effect and inhibitory control (e.g., VanderVeen et al., 2013; Bø et al., 2016), particularly when alcohol-related stimuli were presented (Czapla et al., 2015). Eventually, binge drinkers also demonstrated impairments in sustained attention (Hartley et al., 2004), alerting, and attentional control (Lannoy et al., 2017a). This pattern of cognitive deficits gives support to the continuum hypothesis (e.g., Enoch, 2006; Maurage et al., 2013a), globally suggesting a linear worsening of cognitive dysfunctions in the spectrum of alcohol-related disorders. In this perspective, binge drinking could be considered as a first step toward alcohol-dependence. It has moreover been shown that a subgroup of binge drinkers already had hazardous alcohol consumption associated with negative consequences, identifying them as more likely to develop alcohol-use disorders (Lannoy et al., 2017b). This proposal has been further supported by studies indicating premature brain and cognitive aging in binge drinkers (Sanhueza et al., 2011). Nevertheless, alcohol-dependence is also characterized, beyond cognitive impairments, by large-scale interpersonal and emotional deficits (see Donadon and Osório, 2014 for a review), for which studies are strongly lacking in binge drinking. Understanding emotional information is, however, an essential ability in humans, as it notably allows efficient interpersonal life and social integration. Therefore, this paucity of research and data about emotional processing in binge drinking hampers to have an exhaustive picture of the deficits related to this alcohol consumption pattern and of the continuum hypothesis extension toward non-cognitive factors.

To date, emotional processing has been deeply investigated in alcohol-dependence, showing difficulties in the identification of emotional stimuli from others’ face (Kornreich et al., 2001; Maurage et al., 2008; D’Hondt et al., 2015), voice (Monnot et al., 2002) or body posture (Maurage et al., 2009). This impaired emotional processing among alcohol-dependent individuals seems particularly related to a difficulty for decoding the emotions expressed by others, mainly for negative states (D’Hondt et al., 2014) and with an overestimation of fear (Townshend and Duka, 2003). These emotional deficits are directly associated with difficulties in social interaction, explaining their pivotal role in the emergence and maintenance of alcohol-dependence (Thoma et al., 2013; Oscar-Berman et al., 2014) as emotional-interpersonal problems are an important cause of relapse after detoxification (Zywiak et al., 2003). However, in everyday life, emotional signals are most frequently presented in a crossmodal way (i.e., via the simultaneous presentation of visual and auditory stimuli). Crossmodal integration, defined as the ability to efficiently perceive and integrate sensory signals coming from different modalities in a joint representation, allows the suitable understanding of social and perceptual environment and the generation of an appropriated response (Maurage and Campanella, 2014). In alcohol-dependence, an impaired crossmodal processing of emotions has been identified (Maurage et al., 2007a, 2013b), notably reflected by a disrupted facilitation effect. The facilitation effect is described by a faster processing of congruent crossmodal stimulations compared to unimodal ones, and is classically observed among healthy populations in crossmodal situations (Maurage et al., 2007a). Moreover, this effect is considered as a reliable marker of crossmodal integration in the neurocognitive literature (Calvert et al., 2001). This result thus means that alcohol-dependent patients did not take advantage of the cross-modality to perform emotional decoding. Beyond their impairment for unimodal emotion processing, alcohol-dependent individuals thus present massive deficits in crossmodal ecological situations.

In binge drinking, emotions have scarcely been investigated. Some studies have been interested in the impact of negative emotions on future binge consumption and showed depressive symptoms as a vulnerability factor (Mushquash et al., 2013; Pape and Norström, 2016). Moreover, the co-existence of these two disorders (i.e., binge drinking and depression) leads to more pronounced cognitive deficits (e.g., Hermens et al., 2013b) and specific changes in electrophysiological activity when identifying emotional faces (Connell et al., 2015). Beyond this co-occurrence, some authors have also proposed that this pattern of alternation between excessive alcohol intake and withdrawal episodes induce abnormal neuronal plasticity, in the same way to what is observed in alcohol-dependent patients, and thus would also lead to emotional impairments (Stephens and Duka, 2008). For example, an impaired fear conditioning was observed in student binge drinkers (Stephens et al., 2005) and suggests a reduced ability to adapt behavior in response to aversive events as well as an increased emotional reactivity in situations under which it is not required (e.g., overestimation of negative emotions), as it was previously documented in alcohol-dependence. Impairments for the identification of emotional expressions are furthermore associated with cerebral changes, particularly in the amygdala (Morris et al., 1998). In this perspective, functional modifications in the amygdala are evidenced in binge drinking (Xiao et al., 2013; Campanella et al., 2016). These cerebral impairments could thus further indicate difficulties in the processing of emotional stimuli among binge drinkers. Finally, very few research has investigated emotional processing *per se* in binge drinking. To our knowledge, only one study has examined the behavioral and brain correlates of emotional processing by using vocal stimuli morphed on a continuum between angry and fearful emotions (Maurage et al., 2013a). Results showed that binge drinkers had an impaired identification of emotions together with a reorganization of brain activity (i.e., reduced activation of bilateral superior temporal gyrus and increased activation of right middle frontal gyrus). Overall, these results suggest that binge drinking might also be characterized by impairments in emotional processing. As this research field appears almost unexplored, an in-depth investigation of emotional deficits in binge drinking is needed. This should be made with a more ecological paradigm using crossmodal stimuli, which are the rule rather than the exception in real life social interactions.

The aim of this study was thus twofold: first, to determine whether emotional processing, an essential ability for everyday life social interactions, was altered among student binge drinkers; second, to explore whether the continuum hypothesis, supported for cognitive performance, could be extended toward affective abilities. The current study proposed the exploration of the behavioral performance of binge drinkers and control participants in an emotion detection task implying (a) two emotions differing in their valence (happiness and anger), (b) two modalities of emotional processing (visual and auditory), and (c) the crossmodal integration, further investigated by the facilitation effect. Moreover, as binge drinking was previously associated with inhibition deficits, this study also evaluated the crossmodal inhibition effect, by using an incongruent condition (i.e., requiring to inhibit the interference presented in one of the two modalities). Regarding the continuum perspective, we hypothesized a specific impairment for the facilitation effect in binge drinkers as well as a reduced ability to inhibit non-pertinent modality in incongruent crossmodal situations.

## Materials and Methods

### Participants and Procedure

Participants were recruited through a preliminary anonymous screening, sent by email to all students from the Université catholique de Louvain (Belgium) and 3014 answers were collected. The first part assessed sociodemographic (age, gender, education level, and native language) and psychological variables. The previous or current presence of several disorders (i.e., medical, psychological, neurological, substances consumption, family history of alcohol-dependence) was measured by dichotomous choices (Yes/No) and participants had to specify their response in an open question only if they answered “yes” to the initial item. Second, alcohol consumption in the last 6 months was evaluated by the mean number of alcohol units per drinking occasion, the mean number of drinking occasions per week, the consumption speed (the number of alcohol units consumed per hour), the mean number of alcohol units per week, the drunkenness frequency (by stating that drunkenness refers to loss of coordination, nausea, and/or inability to speak clearly), and the percentage of drunkenness episodes [i.e., (number of drunkenness episodes/total number of drinking occasions)^∗^100]; an alcohol unit corresponding to 10 g of pure ethanol. Finally, drinking motives were measured by targeting four motivations to drink alcohol (i.e., social order, referring to social context; enhancement, referring to the entertaining sensations provoked by alcohol; coping, referring to negative affect regulation; and conformity, referring to others’ negative judgments avoidance; Grant et al., 2007). Participants selected for the study fulfilled the following criteria: native or fluent French speakers, at least 18 years old, no alcohol-dependence and no family history of alcohol-dependence, no positive psychological or neurological disorders, no current medication, no major medical problems, corrected-to-normal visual abilities, normal auditory abilities, total absence of past or current drug consumption (except alcohol and tobacco). On this basis, 120 undergraduate students were contacted, and 40 accepted to take part in the study: 20 Binge Drinkers, recruited according to a binge drinking score (Townshend and Duka, 2005) focusing on the consumption speed and drunkenness frequency (BD; score ≥ 16), and 20 Control Participants (CP; score ≤ 12). The group selection was first conducted according to the binge drinking score because, beyond its frequent use in the literature (e.g., Czapla et al., 2015), it allows targeting the specific binge drinking characteristics (e.g., drink quickly to become rapidly intoxicated). However, to ensure a correct classification of binge drinkers and non-binge drinkers, group comparisons were also performed on all alcohol variables (**Table 1**), which clearly supported the distinction between groups regarding alcohol consumption and binge drinking pattern. All participants (22 women) were aged between 18 and 23 years old (*M* = 19.73, *SD* = 1.74). Before the experiment, participants filled in questionnaires assessing state-trait anxiety (State-Trait Anxiety Inventory, STAI; Spielberger et al., 1983), depression (Beck Depression Inventory, BDI-II; Beck et al., 1996), and alcohol-related disorders (Alcohol Use Disorder Identification Test, AUDIT; Babor et al., 2001). The AUDIT is a 10-item questionnaire, developed by the World Health Organization, evaluating the general harmfulness of alcohol consumption. This test is widely used in the alcohol field and is also considered as a good measure of hazardous alcohol habits in university students (Kokotailo et al., 2004). Participants received a compensation of 10€ for their participation. The study protocol was approved by the ethics committee of the Université catholique de Louvain, and carried out according to the Declaration of Helsinki.

**Table 1:** Demographic and psychological measures for Binge Drinkers (BD) and Control Participants (CP): mean (*SD*).

Variable		BD (*n* = 20)	CP (*n* = 20)
**Demographic measures**			
	Age^ns^	19.65 (1.79)	19.80 (1.74)
	Gender ratio (female/male)^ns^	11/9	11/9
**Psychological measures**			
	Beck depression inventory^ns^	4.35 (3.23)	5.35 (3.54)
	STAI state anxiety inventory^ns^	29.65 (6.44)	33.80 (8.36)
	STAI trait anxiety inventory^ns^	34.90 (6.91)	38.05 (8.67)
**Alcohol consumption measures**			
	Alcohol Use Disorder Identification Test^∗^	16.90 (5.05)	1.10 (2.25)
	Total alcohol units per week^∗^	26.60 (12.12)	1.30 (5.13)
	Number of occasions per week^∗^	3.15 (0.99)	0.30 (0.92)
	Number of alcohol units per occasion^∗^	8.16 (3.54)	0.44 (1.34)
	Consumption speed (units per hour)^∗^	3.35 (1.09)	0.60 (0.54)
**Drinking motives**			
	Enhancement^∗^	16.25 (3.40)	6.26 (1.99)
	Social order^∗^	17.40 (3.36)	8.74 (3.98)
	Conformity^ns^	6.05 (1.39)	6.21 (3.11)
	Coping^∗^	10.10 (3.31)	6.84 (0.50)

^ns^ = *Non-significant*; ^∗^*p < 0.001*.

### Stimuli and Task Description

The emotional crossmodal task assessed emotional detection from emotional facial and vocal stimuli, in separate (unimodal) or simultaneous (crossmodal) ways, the crossmodal conditions presenting identical (crossmodal congruent; e.g., a happy face with a happy voice) or opposite (crossmodal incongruent; e.g., a happy face with an angry voice) emotions. Participants were in a quiet room and placed at 60 cm from the screen. They had to decide as quickly and accurately as possible the emotional content displayed by pressing the appropriate response key with their dominant hand (i.e., 1 for happiness and 2 for anger).

Visual stimuli represented facial expressions of happiness and anger and were selected from the Radboud Faces Database (RaFD; Langner et al., 2010). Vocal stimuli produced vocalizations without semantic content (i.e., the onomatopoeia ≪ Ah ≫) and were selected from a battery of vocal emotional expressions (Maurage et al., 2007b). As visual stimuli led to faster reaction times (RT) than auditory ones (e.g., Joassin et al., 2004), a morphing strategy (i.e., morph between happiness and anger; Morph 2.5., Gryphon Software Corp.) was used in order to obtain similar difficulty in the two unimodal conditions (face and voice), which is a necessary requisite to observe a facilitation effect in the crossmodal congruent condition. Based on a pretest phase (*n* = 11), the morphing level 40–60 was chosen because it led to similar RT in visual and auditory conditions, both for happiness [*t*(10) = 0.67, *p* = 0.517] and anger [*t*(10) = 0.57, *p* = 0.583]. The morphing level was thus set at 60% happiness – 40% anger for happiness faces and 40% happiness – 60% anger for anger faces. The task finally included five men and five women faces as well as five male and five female voices, both depicting happiness and anger.

**FIGURE 1:**
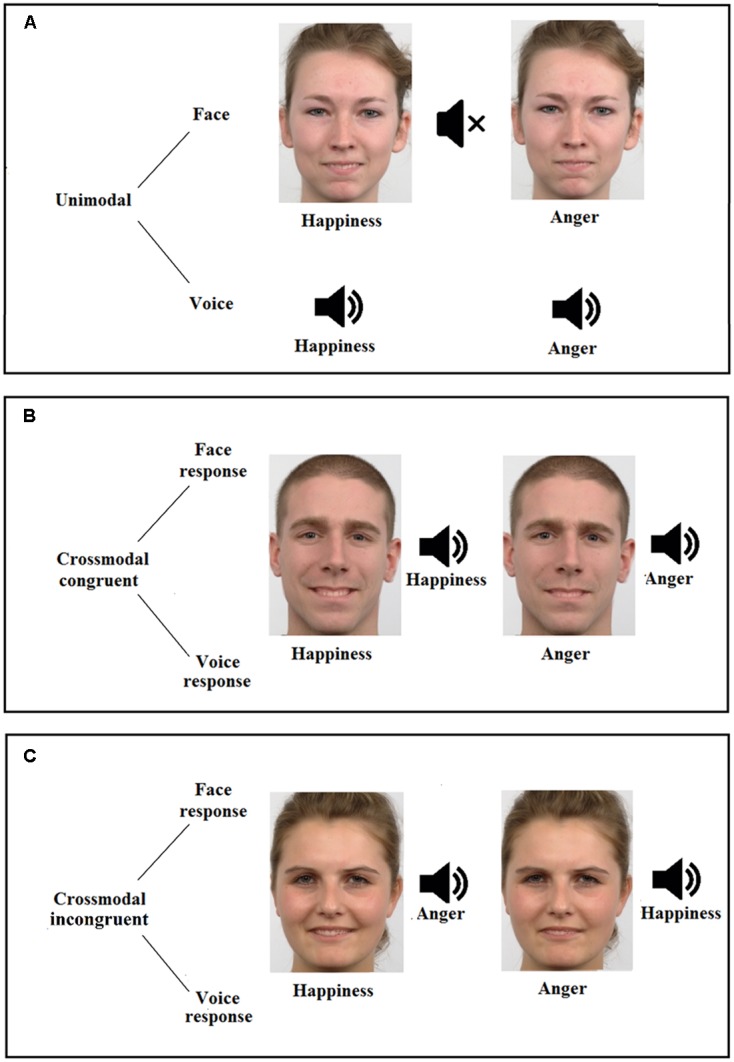
Description of the emotion detection task, displaying the three possible conditions (**A**, unimodal; **B**, crossmodal congruent; and **C**, crossmodal incongruent), the two modalities (face and voice) and the two emotions (happiness and anger). Figure also illustrates two examples of female faces (conditions **A,C**) and an example of male face (condition **B**), for both happiness (morphed with 60% of happiness and 40% of anger, Left) and anger (morphed with 60% of anger and 40% of happiness, Right) faces.

The task comprised 200 unimodal trials (i.e., 100 faces, 100 voices), 200 crossmodal congruent trials (i.e., 100 where the instruction was to focus on the face to answer, 100 where the instruction was to focus on the voice), and 200 crossmodal incongruent trials (i.e., 100 where the instruction was to focus on the face to answer, 100 where the instruction was to focus on the voice). The experimental paradigm was distributed in 3 conditions (unimodal, crossmodal congruent, crossmodal incongruent) × 2 modalities (face, voice) × 2 emotions (happiness, anger) (**Figure 1**). A total of 600 trials were displayed into three blocks (i.e., face unimodal, voice unimodal, and crossmodal), the two first blocks being presented with pseudo-randomized order across participants whereas the experiment always ended with the crossmodal condition. Each trial started with a fixation cross presented for 500 ms, then the stimulus was presented (face, voice, or both) for another 500 ms and followed by a blank screen for 2000 ms. From the stimulus onset, participants thus had 2500 ms to answer. Accuracy Scores (AS; percentage of correct responses) and RT were recorded. Only correct responses were considered for the RT analyses.

### Statistical Analyses

All statistical analyses were performed using SPSS software package (version 21.0) and the significance was set at an alpha level of 0.05. Comparisons between groups were first performed on demographic, psychological, and alcohol consumption characteristics. Then, performance in the emotion detection task were compared via 2 × 2 × 2 × 3 repeated measures analyses of variance (ANOVAs) with Group (CP and BD) as between-subjects factor and Emotion (Happiness and Anger), Modality (Face and Voice), and Condition (Unimodal, Crossmodal Congruent, and Crossmodal Incongruent) as within-subjects factors, computed separately for AS and RT. Finally, bivariate correlations analyses, corrected for multiple comparisons (i.e., Bonferroni’s correction), were performed between task performance and alcohol-related variables (i.e., binge drinking score, AUDIT score, and drinking motives), separately for BD and CP.

## Results

### Demographic and Psychological Measures

Characteristics of each group are reported in **Table 1**. No significant group differences were found for age [*t*(38) = 0.27, *p* = 0.789], gender [χ^2^(1, *N* = 40) = 0, *p* = 1], depressive symptoms [*t*(38) = 0.93, *p* = 0.357], and anxiety (state: [*t*(38) = 1.76, *p* = 0.087], trait: [*t*(38) = 1.27, *p* = 0.211]). Groups, however, significantly differed on all alcohol-related variables, including three of the four drinking motives (i.e., enhancement, social order, and coping).

### Behavioral Analyses

Mean performance and RT for each experimental condition are reported in **Table 2**.

**Table 2:** Accuracy Scores (AS; percentage of correct answers) and Reaction Times (RT; in milliseconds) for Binge Drinkers (BD) and Control Participants (CP) in each experimental condition (i.e., emotions, modalities, and conditions) of the crossmodal emotional identification task: mean (*SD*).

					Conditions	

Emotion	Modality	Variable	Group	Unimodal	Crossmodal congruent	Crossmodal incongruent
Happiness						
	Face	AS	BD	79.10 (12.39)	82.10 (13.05)	78.80 (13.05)
			CP	82.10 (10.89)	79.60 (14.62)	79.20 (13.79)
		RT	BD	873.39 (240.28)	450.96 (113.97)	463 (139.39)
			CP	1057.96 (289.98)	422.62 (100.35)	441.50 (101.64)
	Voice	AS	BD	95.40 (6.30)	91.10 (12.15)	87.50 (8.70)
			CP	95.20 (5.75)	89.10 (10.98)	86.10 (10.89)
		RT	BD	456.55 (182.97)	457.66 (145.82)	465.27 (101.15)
			CP	467.74 (115.73)	432.34 (112.09)	483.59 (150.92)
Anger						
	Face	AS	BD	76.10 (10.47)	71.30 (12.64)	66.50 (12.29)
			CP	74.20 (10.58)	68.90 (16.22)	58.70 (15.73)
		RT	BD	873.65 (238.25)	479.56 (138.96)	477.98 (116.86)
			CP	1096.81 (288.57)	470.34 (119.97)	457.28 (88.44)
	Voice	AS	BD	95.90 (5.13)	92.30 (7.32)	92.10 (8.01)
			CP	96.60 (4.41)	91.70 (8.09)	91.30 (8.34)
		RT	BD	467.29 (146.95)	457.98 (89.33)	476.53 (107.07)
			CP	468.46 (104.40)	441.95 (89.54)	446.51 (104.97)

#### Accuracy Score

Three main effects were identified: Emotion [*F*(1,38) = 7.39, *p* = 0.010, $\eta_{\text{p}}^{2}=0.163$], happiness leading to higher accuracies than anger; Modality [*F*(1,38) = 194.90, *p* < 0.001, $\eta_{\text{p}}^{2}=0.837$], voices leading to better performance than faces; Condition [*F*(2,76) = 21.86, *p* < 0.001, $\eta_{\text{p}}^{2}=0.365$], unimodal trials leading to better accuracies than crossmodal congruent [*t*(39) = 2.79, *p* = 0.008] and crossmodal incongruent [*t*(39) = 6.27, *p* < 0.001] ones, and crossmodal congruent trials leading to better accuracies than incongruent trials [*t*(39) = 5.28, *p* < 0.001]. An interaction between Emotion and Modality [*F*(1,38) = 29.92, *p* < 0.001, $\eta_{\text{p}}^{2}=0.440$] was also found. These effects were qualified by a triple interaction between Emotion, Modality, and Condition [*F*(2,76) = 17.34, *p* < 0.001, $\eta_{\text{p}}^{2}=0.313$]. In the unimodal Condition, there was no difference in the identification of happiness and anger, both for face [*t*(39) = 1.92, *p* = 0.063] and voice [*t*(39) = 0.76, *p* = 0.443] modalities. In the crossmodal congruent Condition, happiness was better identified than anger in the face Modality [*t*(39) = 3.79, *p* = 0.001] but not in the voice Modality [*t*(39) = 1.33, *p* = 0.191]. In the crossmodal incongruent Condition, happiness was better recognized than anger in the face Modality [*t*(39) = 5.82, *p* < 0.001] but anger was better identified in the voice Modality [*t*(39) = 2.95, *p* = 0.005]. There was no interaction effect between Emotion and Condition [*F*(2,76) = 2.88, *p* = 0.062, $\eta_{\text{p}}^{2}=0.070$] or Modality and Condition [*F*(2,76) = 2.74, *p* = 0.071, $\eta_{\text{p}}^{2}=0.067$]. Moreover, and centrally, there was no main Group effect [*F*(1,38) = 0.49, *p* = 0.490, $\eta_{\text{p}}^{2}=0.013$] nor any interaction between Emotion and Group [*F*(1,38) = 0.31, *p* = 0.584, $\eta_{\text{p}}^{2}=0.008$]; Modality and Group [*F*(1,38) = 0.22, *p* = 0.645, $\eta_{\text{p}}^{2}=0.006$]; Condition and Group [*F*(2,76) = 1.05, *p* = 0.356, $\eta_{\text{p}}^{2}=0.027$]; Emotion, Modality, and Group [*F*(1,38) = 1.16, *p* = 0.288, $\eta_{\text{p}}^{2}=0.030$]; Emotion, Condition, and Group [*F*(1,76) = 1.21, *p* = 0.303, $\eta_{\text{p}}^{2}=0.031$]; Modality, Condition, and Group [*F*(2,76) = 0.62, *p* = 0.542, $\eta_{\text{p}}^{2}=0.016$]; as well as Emotion, Modality, Condition, and Group [*F*(2,76) = 1.10, *p* = 0.337, $\eta_{\text{p}}^{2}=0.028$].

#### Reaction Times

**FIGURE 2:**
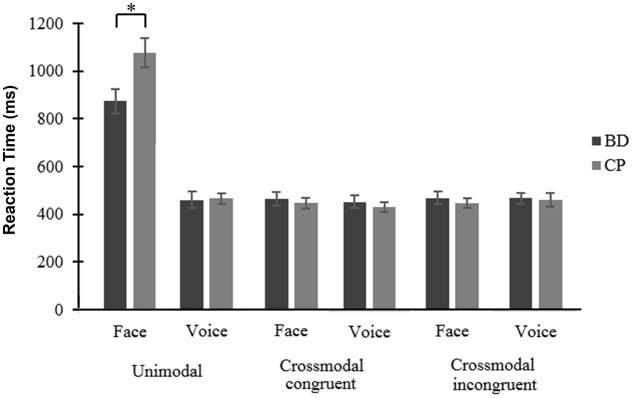
Reaction times (in milliseconds) for the two modalities (face and voice) of emotional processing, within the three possible conditions (unimodal, crossmodal congruent, and crossmodal incongruent) among Binge Drinkers (BD) and Control Participants (CP). ^∗^*p* < 0.05. Bars represent the mean value for each condition and whiskers represent the standard error.

While there was no main effect of Emotion [*F*(1,38) = 3.53, *p* = 0.068, $\eta_{\text{p}}^{2}=0.085$], results showed a main effect of Modality [*F*(1,38) = 117.70, *p* < 0.001, $\eta_{\text{p}}^{2}=0.756$], voices leading to faster processing than faces and a main effect of Condition [*F*(2,76) = 116.12, *p* < 0.001, $\eta_{\text{p}}^{2}=0.753$], crossmodal congruent trials leading to faster processing than crossmodal incongruent [*t*(39) = 2.37, *p* = 0.023] and unimodal [*t*(39) = 10.27, *p* < 0.001] trials, and crossmodal incongruent trials leading to faster processing than unimodal trials [*t*(39) = 10.16, *p* < 0.001]. These effects were qualified by two interactions between Condition and Group [*F*(2,76) = 6.24, *p* = 0.003, $\eta_{\text{p}}^{2}=0.141$], and between Modality, Condition, and Group [*F*(2,76) = 6.88, *p* = 0.002, $\eta_{\text{p}}^{2}=0.153$]. First, conditions comparison showed no significant difference between groups (all *p* ≥ 0.062); in both groups, crossmodal Condition led to faster RT than unimodal ones, but this difference was larger in CP than in BD [i.e., for congruent trials, *t*(19) = 2.46, *p* = 0.024, and for incongruent trials, *t*(19) = 2.50, *p* = 0.022]. Second, the triple interaction showed a faster processing of face unimodal trials in BD compared to CP [*t*(38) = 2.47, *p* = 0.018] (**Figure 2**). An interaction was also found between Emotion and Modality [*F*(1,38) = 4.97, *p* = 0.032, $\eta_{\text{p}}^{2}=0.116$], showing that happiness processing was faster than anger processing for faces [*t*(39) = 2.95, *p* = 0.005] while no significant difference was found for voices [*t*(39) = 0.85, *p* = 0.932]. Finally, an interaction between Modality and Condition [*F*(2,76) = 170.86, *p* < 0.001, $\eta_{\text{p}}^{2}=0.818$] showed that voice processing was faster than face processing in unimodal Conditions [*t*(39) = 13, *p* < 0.001]. However, there was no significant difference for crossmodal congruent [*t*(39) = 0.55, *p* = 0.583] and incongruent [*t*(39) = 0.58, *p* = 0.565] conditions. No main group effect was found [*F*(1,38) = 0.52, *p* = 0.477, $\eta_{\text{p}}^{2}=0.013$] nor any interaction between Emotion and Group [*F*(1,38) = 0.02, *p* = 0.901, η_p_^2^ = 0]; Modality and Group [*F*(1,38) = 3.83, *p* = 0.058, $\eta_{\text{p}}^{2}=0.092$]; Emotion and Condition [*F*(2,76) = 1.62, *p* = 0.205, $\eta_{\text{p}}^{2}=0.041$]; Emotion, Modality, and Condition [*F*(2,76) = 0.32, *p* = 0.730, $\eta_{\text{p}}^{2}=0.008$]; Emotion, Modality, and Group [*F*(1,38) = 2.54, *p* = 0.120, $\eta_{\text{p}}^{2}=0.063$]; Emotion, Condition, and Group [*F*(2,76) = 1.88, *p* = 0.160, $\eta_{\text{p}}^{2}=0.047$]; as well as Emotion, Modality, Condition, and Group [*F*(2,76) = 0.40, *p* = 0.675, $\eta_{\text{p}}^{2}=0.010$].

### Correlational Analyses

First, correlations analyses conducted between emotional processing abilities (AS and RT) and alcohol consumption characteristics, using binge drinking and AUDIT scores, showed no significant relationship (all *p* > 0.05). Second, correlations between emotional processing abilities and drinking motives were not significant for social order, conformity, and coping motives (all *p* > 0.05). However, significant correlations were found in BD group between enhancement motive and the percentage of correct anger identification in face crossmodal congruent trials (*r* = 0.77, *p* = 0.003) and face crossmodal incongruent trials (*r* = 0.70, *p* = 0.048). The presented *p*-values were adjusted after Bonferroni correction.

## Discussion

The aims of this study were to evaluate emotional processing among binge drinkers and to explore the extension of the continuum hypothesis toward affective abilities. Indeed, while earlier studies have underlined a wide range of interpersonal and emotional impairments in alcohol-dependence (notably for crossmodal processing), no available data using more ecological paradigms allowed determining whether binge drinking, potentially considered as a first step toward alcohol-dependence, was also characterized by emotional impairments. For this purpose, the performance of binge drinkers and controls was compared during an emotion detection task using crossmodal stimuli which are characteristic of the everyday life interactions, particularly in emotional context.

On the one hand, this study reveals that BD are not impaired for the processing of emotional stimuli, centrally showing that the emotional difficulties widely described in alcohol-dependence do not constitute a central deficit at the early stages of alcohol-related disorders. Actually, BD even appeared faster than CP for the detection of emotional facial expressions in unimodal condition. A hypothesis to understand this finding could be made through the perception of social context in undergraduate students. Research focusing on emotions has indeed largely underlined that emotions are innately social (van Kleef et al., 2016). Indeed, emotional expression is based on the perception of other’s emotions or social context and thus appears as a response to other people or social norms (Fischer and van Kleef, 2010). Therefore, it has been shown that social factors influence (e.g., Stamkou et al., 2016) and even improve (Bublatzky et al., 2014) the recognition and interpretation of facial emotional expressions. Besides, regarding alcohol consumption in youth, longitudinal study targeting people from adolescence to adulthood showed greater social acceptance and social integration in alcohol users, including binge drinkers (Pedersen and von Soest, 2015). Taken together, these results suggest that a more efficient social environment in BD might be related to the faster emotional detection observed in this group in the current study. This social context can be understood by the specific motivations related to alcohol in young drinkers (e.g., because it is fun or exciting) and notably by the motivations associated with social interactions (e.g., to feel more relax). These motivations involve alcohol expectancies and drinking motives, both being strongly relevant in binge drinking (e.g., Van Tyne et al., 2012). In this respect, the current study showed that BD had significantly greater drinking motives associated to enhancement, social order, and coping. Especially, among BD, a positive relationship was found between enhancement and the correct anger identification in face crossmodal conditions. In other words, it suggests that the more BD drink alcohol for positive reinforcement and agreeable sensations, which is frequently related to social context in undergraduates, the more they are effective to recognize emotional facial expressions of anger in crossmodal condition. It could thus be hypothesized that student BD with higher enhancement motives and drinking alcohol for positive reinforcement, notably in social situations, improve their ability to recognize others’ emotions through a repeated and prolonged involvement in social context in comparison to students being less socially involved. Moreover, these findings highlight that social environment is very different in binge drinking than in alcohol-dependence, rather described as a disorder related to social isolation, which suggest that the continuum hypothesis could not be applied to emotional processing. However, it is also important to underline that binge drinking is not defined as a unitary group, also implying that the BD who could evolve toward alcohol-dependence represent a specific subgroup (e.g., Lannoy et al., 2017b). Hazardous BD, characterized by greater alcohol consumption associated with strong negative consequences, could therefore present impaired emotional processing, while more recreational BD (characterized by heavy alcohol use but less negative consequences including in self-reported control abilities) could present preserved emotional abilities.

On the other hand, the classical effects found in crossmodal tasks were observed in this study. First, results indicate a facilitation effect in both groups, characterized by a faster processing of crossmodal congruent than unimodal trials. This effect was more pronounced in the control group, however, as CP was slower than BD to identify facial expressions in unimodal condition, the current results cannot suggest an impaired facilitation effect among BD. Indeed, this greater difference between unimodal and crossmodal congruent trials in CP could be rather explained by a slower processing of face unimodal condition. These results are thus in line with the discussed hypothesis concerning social context, as the facilitation effect is typically related to the correct integration of social environment in different modalities. Second, an interference effect was also shown in both groups, indexed by better AS and faster RT in crossmodal congruent than incongruent trials. Moreover, findings put forward that this effect was similar in CP and BD, suggesting that BD correctly inhibit the interference from the incongruent modality. Therefore, while inhibition of interference has been identified as a reliable predictor of binge drinking (Paz et al., 2016) and found to be impaired in this population using tasks probing attentional networks (Lannoy et al., 2017a), the paradigm used in this study required to focus on one modality (and therefore inhibit the other) during a half block with no instruction change. It thus appears easier than classical inhibition tasks and could explain the good performance of BD.

Finally, this study presents some limitations. First, even if previous studies have asserted that face stimuli should be modified to have the same complexity than voice stimuli (e.g., Joassin et al., 2004), and whereas the current pretest phase highlighted an optimal morphing level at 40–60, as it was also used in previous studies (e.g., Maurage et al., 2007a), voice unimodal trials led to faster processing and better accuracies than faces, suggesting that future studies should confirm the use of this morphing level and potentially determine a more efficient level of complexity. Second, some variables used in this study to assess alcohol consumption appear quite subjective (e.g., the drunkenness). While group selection and statistical analyses support the consistency between all alcohol measures (those used to compute binge drinking score and those used to evaluate the number of drinks consumed), it should be underlined and taken into account in future studies.

This first exploration of emotional processing in binge drinking did not allow to highlight group differences and thus suggests preserved emotional detection and crossmodal integration among BD. In previous studies, the abnormal cerebral activity leading to emotional processing impairments was identified as the result of numerous withdrawals (Duka et al., 2004) and relapses in alcohol-dependent patients. This argument led to the proposal that binge drinking pattern, especially characterized by the alternation between intense intake and abstinence periods, would also be associated with emotional impairments (Stephens and Duka, 2008). The current study, however, conveys that basic emotional processing is preserved at the first stages of alcohol-related disorders and that these impairments could rather appear in the transition between binge drinking and alcohol-dependence. Indeed, the earlier identification of impaired emotional detection (Maurage et al., 2013a) used more complex vocal stimuli presenting different morphing levels between angry and fearful rather than one positive and one negative emotional content, always presented with the same complexity. Nevertheless, considering the main advantages of crossmodal explorations (Maurage and Campanella, 2014), the identification of specific brain correlates dedicated to crossmodal integration (Maurage et al., 2013b), and the results found in alcohol-dependence, neuroscientific approaches could be useful to highlight possible cerebral alterations during crossmodal processing in binge drinking. Neuroscience studies indeed allow for underlining cerebral changes before the emergence of behavioral deficits, and have brought valuable contributions to the binge drinking research field (see Hermens et al., 2013a; Maurage et al., 2013c for reviews). It might be hypothesized that the preserved behavioral performance observed here actually masks underlying subtle brain modifications.

## Conclusion

While this preliminary investigation of emotional processing in binge drinking did not emphasize difficulty for emotional detection or crossmodal integration, it bares central perspectives for future studies. Indeed, as one previous study had identified emotional deficits at the behavioral and brain levels in binge drinking, it suggests that emotional abilities are not totally preserved when complex emotional decoding is requested (Maurage et al., 2013a). The current study thus contributes to specifying that the impairments presented by BD depend on the nature and the complexity of the evaluation. Moreover, the ecological design using crossmodal stimuli brings light to the potential beneficial features associated with binge drinking (e.g., positive motivations and social integration), underlining its main distinction with alcohol-dependence, as BD appears preserved in close to real life crossmodal situations. Primary emotional detection thus seems to be preserved, indicating that BD would be undermined only in more complicated situations. Finally, these results suggest that the continuum hypothesis cannot be generalized toward the broad field of emotions processing, and urge future studies to deepen the exploration of emotional and cognitive abilities in binge drinking. A precise description of the impaired versus preserved abilities characterizing this alcohol consumption pattern is needed to have a clearer view of the extent and limits of the continuum hypothesis.

## Author Contributions

SL, MB, and PM developed the study. All authors contributed to the study design. Data collection was conducted by SL and data analyses were performed in collaboration with all authors (SL, VD, MB, JB, and PM). SL drafted the paper under the supervision of PM, while VD, MB, and JB provided critical revisions. All authors approved the final version of the paper.

## Conflict of Interest Statement

The authors declare that the research was conducted in the absence of any commercial or financial relationships that could be construed as a potential conflict of interest.
